# Visual genetic typing of glioma using proximity‐anchored in situ spectral coding amplification

**DOI:** 10.1002/EXP.20220175

**Published:** 2023-07-06

**Authors:** Xiaolei Chen, Ruijie Deng, Dongdong Su, Xiaochen Ma, Xu Han, Shizheng Wang, Yuqing Xia, Zifu Yang, Ningqiang Gong, Yanwei Jia, Xueyun Gao, Xiaojun Ren

**Affiliations:** ^1^ Department of Chemistry and Biology Faculty of Environment and Life Science Beijing University of Technology Beijing China; ^2^ College of Biomass Science and Engineering Healthy Food Evaluation Research Center Sichuan University Chengdu China; ^3^ Institute of High Energy Physics Chinese Academy of Sciences Beijing China; ^4^ Department of Bioengineering University of Pennsylvania Philadelphia USA; ^5^ State‐Key Laboratory of Analog and Mixed‐Signal VLSI Institute of Microelectronics University of Macau Macau China

**Keywords:** genetic typing, glioma, in situ imaging, multiplexed detection, RNA mutation

## Abstract

Gliomas are histologically and genetically heterogeneous tumors. However, classical histopathological typing often ignores the high heterogeneity of tumors and thus cannot meet the requirements of precise pathological diagnosis. Here, proximity‐anchored in situ spectral coding amplification (ProxISCA) is proposed for multiplexed imaging of RNA mutations, enabling visual typing of brain gliomas with different pathological grades at the single‐cell and tissue levels. The ligation‐based padlock probe can discriminate one‐nucleotide variations, and the design of proximity primers enables the anchoring of amplicons on target RNA, thus improving localization accuracy. The DNA module‐based spectral coding strategy can dramatically improve the multiplexing capacity for imaging RNA mutations through one‐time labelling, with low cost and simple operation. One‐target‐one‐amplicon amplification confers ProxISCA the ability to quantify RNA mutation copy number with single‐molecule resolution. Based on this approach, it is found that gliomas with higher malignant grades express more genes with high correlation at the cellular and tissue levels and show greater cellular heterogeneity. ProxISCA provides a tool for glioma research and precise diagnosis, which can reveal the relationship between cellular heterogeneity and glioma occurrence or development and assist in pathological prognosis.

## Graphical abstract

This research developed a novel multiplexed imaging method of RNA mutations, named proximity‐anchored in situ spectral coding amplification (ProxISCA), enabling visual typing of brain gliomas with different pathological grades at the single‐cell and tissue levels. ProxISCA provides a tool for glioma research and precise diagnosis, which can reveal the relationship between cellular heterogeneity and glioma occurrence or development, and assist in pathological prognosis.

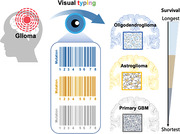

## 1 INTRODUCTION

Gliomas, the most common primary intracranial tumors, are histologically and genetically heterogeneous.^[^ ^1^ ^]^ Tumor cell heterogeneity may originate from cell cycle heterogeneity, cell microenvironment heterogeneity, and stochasticity of gene expression.^[^ ^2^, ^3^ ^]^ These heterogeneities may have functional effects on cell phenotypes, leading to differences in tumor proliferative capacity, invasive capacity, and drug sensitivity, which ultimately affect the diagnosis and treatment of cancer patients.^[^ ^4^, ^5^, ^6^ ^]^ Classic histopathological classification often ignores the heterogeneity of malignant tumors and cannot effectively guide the diagnosis and prognosis of patients.^[^ ^7^, ^8^ ^]^ Gliomas with the same pathological morphology are highly heterogeneous at the molecular level due to molecular genetic changes, resulting in very different tumor prognoses and responses to treatment.^[^ ^9^, ^10^ ^]^ Thus, genotype‐based glioma typing can guide the individualized diagnosis and treatment of patients more objectively and reasonably.^[^ ^11^, ^12^ ^]^ WHO 2016 indicated that a series of molecular markers were helpful in the clinical diagnosis and prognosis of gliomas.^[^ ^13^, ^14^ ^]^ Among them, the mutation status of isocitrate dehydrogenase (IDH1)^[^ ^15^, ^16^ ^]^/telomerase reverse transcriptase (TERT)^[^ ^17^, ^18^ ^]^/alpha thalassemia/mental retardation syndrome X‐linked (ATRX),^[^ ^19^ ^]^ and deletion status of chromosomes 1p and 19q^[^ ^20^, ^21^ ^]^ are usually combined for molecular typing and prognosis of gliomas with different pathological grades.^[^ ^22^, ^23^ ^]^

Conventional detection of multiple gene mutations is often realized by genome sequencing, which can offer the average value of gene expression in a group of cells.^[^ ^25^, ^26^ ^]^ It is difficult to identify the genetic signatures of various cell subsets or a few key cells in a heterogeneous population. Single‐cell RNA sequencing (scRNA‐seq) can obtain information at the single‐cell level and identify various cell types on the basis of gene expression profiles.^[^ ^27^, ^28^ ^]^ However, this method has relatively low detection efficiency and loses the spatial location information of cells. In situ rolling circle amplification (in situ RCA) is a promising method for imaging RNA with high specificity at single‐molecule resolution, enabling local isothermal amplification and providing localization information of target molecules. In addition, the technique can identify single‐base mutations in target sequences, making it particularly suitable to detect single point mutations and single nucleotide polymorphisms.^[^ ^29^, ^30^, ^31^ ^]^ However, developed RCA‐based methods often have low detection throughput and potentially suffer from spatial localization inaccuracy as the amplification product detaches from the target RNA. Fluorescent in situ sequencing (FISSEQ) advanced RCA methods and enabled high‐throughput multiplexed imaging, but it inevitably led to consumed time, high cost, weakened labelling signal, and the increased error rate of base calling due to multiple labelling processes.^[^ ^32^, ^33^ ^]^ Multiplexed error‐robust fluorescence in situ hybridization (MERFISH) employed combination label, continuous imaging, and binary barcode technology to significantly improve the detection throughput while greatly reducing costs and detection errors. However, this method has difficulty achieving single‐base recognition of RNA.^[^ ^34^, ^35^ ^]^ Thus, tools to identify the single‐base mutation and its subcellular spatial location with a multiplexing capacity are in high demand for accurate glioma typing but are still challenging.

In this work, we designed a proximity‐anchored in situ spectral coding amplification (ProxISCA) strategy, enabling imaging of eight RNA mutations or RNA‐related markers in glioma cells at single‐molecule resolution. The designed ligation‐based padlock probe can specifically recognize the mutation site, and the proximity primer enabled anchoring of the amplicon on the target RNA, thus improving localization accuracy. By constructing a DNA module‐based spectral coding strategy, multiple RNA mutations can be imaged with low cost and simple operation through only one round of labelling. Owing to the one‐target‐one amplicon amplification, this method allows the quantification of RNA mutations with single‐molecule resolution. With this method, we measured eight RNA biomarkers of glioma cells and realized in situ typing of oligodendroglioma, astrocytoma/secondary glioblastoma (sGBM), and primary glioblastoma (pGBM) in brain tissue. This method provides a new single‐molecule multithroughput analysis tool for RNA mutations for precise pathological diagnosis to explore the precise relationship between cellular heterogeneity and glioma occurrence/development.

## 2 EXPERIMENTAL METHODS

### 2.1 Materials and reagents

Adenosine triphosphate (ATP), T4 polynucleotide kinase (PNK), T4 DNA ligase, phi29 DNA polymerase, RiboLock RNase Inhibitor (RRI), and deoxyribonucleotides mixture (dNTPs) were purchased from Thermo Fisher Scientific (Waltham, USA). Yeast tRNA was purchased from Ruitaibio Co., Ltd. (Beijing, China). Dithiothreitol (DTT), diethyl pyrocarbonate (DEPC)‐treated H2O, and DEPC‐treated phosphate buffered saline (DEPC‐PBS) were purchased from Beyotime Co., Ltd. (Beijing, China). Salmon sperm DNA was purchased from Beijing Solarbio Science and Technology Co., Ltd. (Beijing, China). The 4% (w/v) paraformaldehyde (PFA) in PBS buffer, 1% gelatin in PBS buffer, Tween‐20, formamide, and 20× saline sodium citrate (SSC) buffer (pH 7.4) were purchased from Leagene Co., Ltd. (Beijing, China). Triton X‐100 was purchased from Sigma‒Aldrich (St. Louis, USA). Fluoromount‐G and Fluoromount‐G with DAPI were purchased from Southern Biotech (Birmingham, USA). TransZol Up, TransScript one‐step gDNA removal, cDNA synthesis, and Green qPCR SuperMix Kit were purchased from TransGene Biotech Co., Ltd. (Beijing, China). 1× PBS, fetal bovine serum, 0.25% Trypsin, 0.05% Trypsin, Dulbecco's modified Eagle's medium (DMEM, high glucose), MEM‐EBSS (Eagle's minimum essential medium with Earle's Balanced Salts), MEM NEAAS (Non‐Essential Amino Acids Solution) (100×) were purchased from Thermo Fisher Scientific (Waltham, USA). Penicillin‒streptomycin (100×) was purchased from New Cell and Molecular Biotech Co., Ltd. (Suzhou, China).

The DNA sequences (Table S1) were purchased from Rui Biotech Co., Ltd. (Beijing, China) and were purified by high‐performance liquid chromatography (HPLC). The fluorophore‐modified probes (Alexa405, Alexa488, Alexa555, and Cy5) were purchased from Invitrogen (Beijing, China) and Sangon Biotech Co., Ltd. (Shanghai, China), and all were HPLC purified. HeLa (human cervical cancer cell), U87MG (human glioma cell), and U251 (human glioma cell) cells were purchased from Peking Union Cell Resource Center (Beijing, China). MO3.13 (human oligodendrocytic cell) was purchased from Bluefbio (Shanghai) Biology Technology Development Co., Ltd. (Shanghai, China). U87MG cells were also purchased from ATCC (U.S. Model Culture Collection Repository). The clinical tissue sections were from the Chinese People's Liberation Army General Hospital.

### 2.2 Cell culture

U87MG cells purchased from Peking Union Cell Resource Center were maintained in MEM‐EBSS medium supplemented with 10% fetal bovine serum and 1% MEM NEAAS (100×). U251 cells were maintained in MEM supplemented with 10% fetal bovine serum. MO3.13, HeLa and A375 cells were maintained in DMEM (high glucose) supplemented with 10% fetal bovine serum and 1% penicillin‒streptomycin (100×). The cells were grown at 37°C, 5% CO_2_, and 95% air humidity.

### 2.3 Cell pretreatment procedure

Cells were seeded on a gelatin‐coated coverglass (22 mm × 22 mm, VWR, USA) enclosed by polydimethylsiloxane (PDMS) with a 3 mm diameter chamber and allowed to grow adherently. The cells were fixed in 4% PFA in 1× PBS buffer for 15 min at 20–25°C when the cells reached the desired confluence. This was followed by two brief washes with 1× DEPC‐treated PBS. After fixation, the cells were permeabilized for 5 min with 1× PBS solution with 0.5% Triton‐X100 at room temperature. Then, the cells were washed twice with 1× DEPC‐treated PBS.

### 2.4 Phosphorylation of the linear padlock probe

The linear padlock probes were first phosphorylated in a 20 μL mixture containing 10 μL of the linear padlock probe (20 μm), 2 μL of ATP (10 mm), 2 μL of 10× T4 PNK reaction buffer, and 0.5 μL of T4 PNK (5 U μL^–1^) at 37°C for 1 h. Then, the phosphorylation reaction was terminated at 65°C for 20 min using a Bio‐Rad T100 Thermal Cycler.

### 2.5 ProxISCA procedure

Hybridization of the padlock probe with the target biomarker was conducted in a 20 μL mixture produced by adding 2 μL of each phosphorylated padlock probe (10 μm), 1 μL of DTT (100 mm), 2 μL of yeast tRNA (10 mg mL^–1^), and 0.5 μL of RRI (40 U μL^–1^) overnight at 37°C. The sample was washed twice for 3 min at room temperature by DEPC‐PBS with 0.05% Tween‐20 (PBS‐T). Then, the hybridization of proximity primer with the target was performed in a 20 μL mixture containing 2 μL of 20× SSC, 2 μL of formamide, 1 μL of DTT (100 mm), 1 μL of proximity primer (4 μm), and 0.5 μL of RRI at 37°C for 60 min, following a wash using PBS‐T. Then, a 10 μL circularization reaction mixture containing 1 μL of 10× T4 ligation buffer, 1 μL of T4 DNA ligase (5 U μL^–1^) and 0.25 μL of RRI was added to the sample and incubated at 37°C for 2 h. In situ amplification was then performed in a 10 μL reaction mixture containing 1 μL of 10× phi29 DNA polymerase reaction buffer, 3 μL of dNTPs (10 mm), 0.5 μL of phi29 DNA polymerase, and 0.25 μL of RRI at 37°C for 2 h. This was followed by a wash using 1× PBS‐T. The amplicons were imaged by hybridization with fluorophore‐modified detection probes (500 nm) in 2× SSC, 4% formamide and salmon sperm DNA (10 ng μL^–1^) at 37°C for 30 min. The sample was washed three times with PBS‐T. The slides with treated samples were ready for visualization after being mounted with Fluoromount‐G. The samples were wrapped in tin foil to protect them from light before testing.

### 2.6 Tissue section treatment procedure

Glioma tissue sections were obtained from the Chinese People's Liberation Army General Hospital and approved to use (Ethical Approval No. 2018‐268‐02). Informed consent was obtained from the sampled patients. Paraffin sections from patients were first dewaxed. The tissue sections were treated sequentially with xylene I for 20 min, xylene II for 20 min, anhydrous ethanol I for 5 min, anhydrous ethanol II for 5 min, and 75% alcohol for 5 min and then washed with ultrapure water or dried naturally. The ProxISCA procedure was similar to that of cells except that the amplification time was extended to 5 h. Moreover, the paraffin sections were placed on small grids in a humidity chamber for incubation, and an appropriate height of DEPC water was added at the bottom of the humidity chamber. The chamber provides a dark, humid environment, which helps avoid the quenching of fluorescent probes and prevents the evaporation of amplification reagents. Finally, the sealed humidity chamber was placed in a heating cabinet for incubation at the corresponding temperature and duration.

### 2.7 Image acquisition and analysis

Fluorescence imaging was carried out by fluorescence confocal microscopy (Nikon, 60× oil immersion lens was used). The Alexa405, Alexa488, Alexa555, and Cy5 dyes were excited with laser lines of 405 nm, Ar+ (488 nm), HeNe543 (543 nm), and HeNe633 (633 nm), respectively, and detected with bandpass filters of 425–475 nm, 500–550 nm, 570–620 nm, and 663–738 nm, respectively. To ensure that all amplicons were visualized, z‐stack images were taken with a distance of 0.4 μm between the z‐slices. The z‐slices were combined into a single image by creating maximum intensity projection using NIS‐Elements AR Analysis 45100. The stitched image of different channels was processed by ImageJ version 1.46r software. The bright fluorescent amplicons were discriminated from the background by setting the offset value. The outline of the cells was determined by bright field images.

### 2.8 Real‐time quantitative PCR (RT‒qPCR) analysis of RNA inside cells

Total RNA was extracted from U87MG cells using TransZol Up following the manufacturer's instructions. The primers involved in the assay are listed in Table S2. The cDNA samples were synthesized by a TransScript one‐step gDNA removal and cDNA synthesis kit. This reaction was performed in a 20 μL mixture containing 2 μL of total RNA (50 ng to 5 μg), 1 μL of reverse primer (1 μm), 1 μL of gDNA remover, 10 μL of 2× TS reaction mix, and 1 μL of TransScript RT/RI enzyme mix at 42°C for 15 min. Then, the reverse transcriptase was heat inactivated for 5 s at 85°C. qPCR analysis of RNA targets was performed with Green qPCR SuperMix on a Roche LightCycler 96. The 20 μL reaction solution was prepared by adding 2 μL of cDNA sample, 1 μL forward primer (10 μm), 1 μL reverse primer (10 μm), and 10 μL 2× Green qPCR SuperMix. The qPCR conditions were as follows: incubation at 94°C for 30 s for the hot start, 94°C for 5 s for annealing, followed by 45 cycles of 55°C for 15 s and 72°C for 10 s. The GAPDH gene served as an internal reference for relative quantitative analysis.

## 3 RESULTS AND DISCUSSION

### 3.1 Principle of the ProxISCA

**SCHEME 1:**
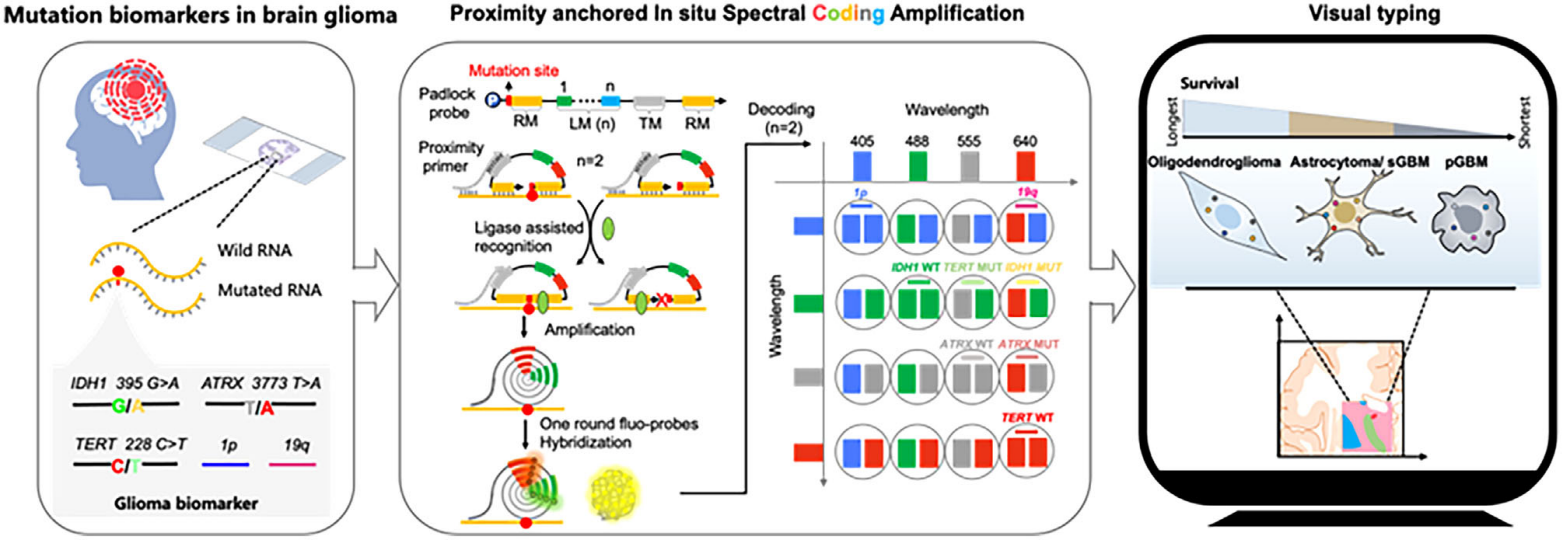
Schematic diagram of ProxISCA for in situ visual typing of gliomas. The scheme illustrates the procedures of the in situ visual typing of different gliomas by ProxISCA. We chose eight biomarkers involved in the typing of different glioma grades, including single base mutations in IDH1 at site 395 (G > A), ATRX at site 3773 (T > A), TERT at site 228 (C > T), the D1S2666 gene on the short arm of chromosome 1 at 36.23 (1p) and the D1S412 gene on the long arm of chromosome 19 at 13.3 (19q). Oligodendroglioma was classified by IDH mutation and codeletion of 1p/19q, accompanied by the expression of TERT MUT and ATRX WT, which have the longest survival. Glioma with the expression of IDH WT, accompanied by mutation of TERT or ATRX, belongs to primary GBM, which has the worst prognosis. Astrocytoma/secondary glioblastoma (sGBM) was classified by expression of IDH MUT, deletion of 1p/19q absent, accompanied by the expression of TERT MUT/ATRX MUT, of which prognosis was between oligodendroglioma and primary GBM.

The padlock probe includes four modules: recognition module (RM), trigger priming module (TM), adjacent module (AM), and labelling module (LM). LM is extensible and can contain multiple different modules. The mutated position is designed at the first base of the 5′‐terminus of the padlock probe, which is directly near the nick enclosed by the ligase. The design can ensure the highest discriminatory capacity of the single mutated base. The proximity primer was designed for simultaneous targeting to the AM and TM, triggering amplification and attaching the amplified product to the target. The padlock probe can specifically recognize the RNA template and be circularized in the presence of T4 DNA ligase. Next, the proximity primer is extended by amplification to produce a long single‐stranded amplified product with hundreds of copies of the complement of the padlock probe. The amplified product forms as a nanoclew, which can be visualized upon the hybridization of fluorophore‐labelled detection probes. The fluorescence coding strategy was constructed by the combination of two tagging modules and four types of fluorescent dye (Alexa 405/Alexa 488/Alexa 555/Cy5)‐labelled probes. The fluorescence barcodes for labelling different targets are presented in Scheme 1. Attributed to the one‐target‐one‐amplicon amplification process, ProxISCA can visualize RNA mutations at single‐molecule resolution.

### 3.2 Multiplex imaging of RNA mutations in single cells by ProxISCA

**FIGURE 1:**
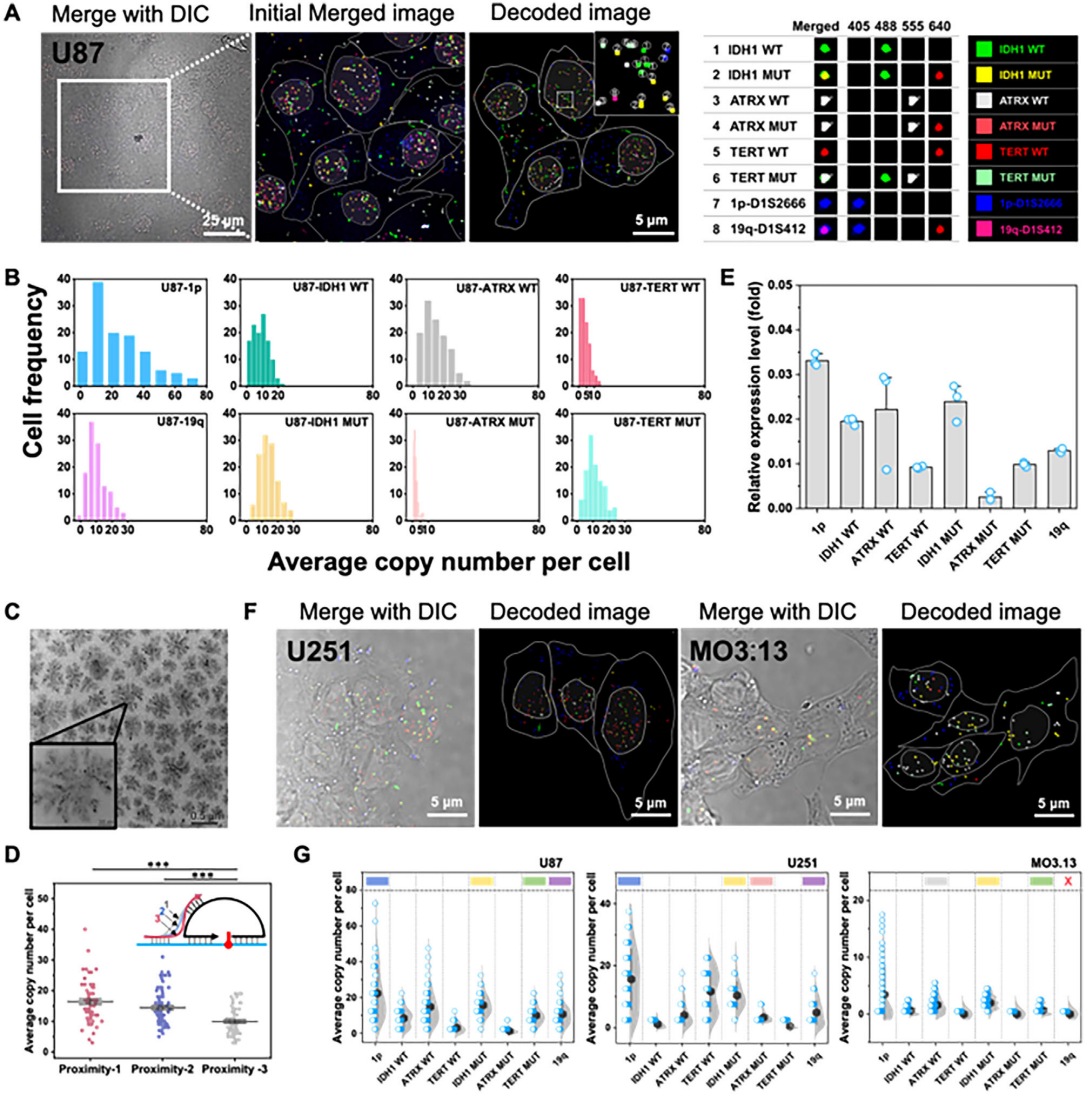
Multiplex imaging of RNA mutations in single cells by ProxISCA. (A) Confocal merged images of the eight target markers imaged by ProxISCA. The decoded amplicons are marked with colored squares based on their coding assignment. Inset: the enlarged fluorescent image with numbered circles corresponding to the encoded RNA mutation molecules. The cell outline is marked with a solid gray line and filled with light gray to indicate the nucleus. The right‐most chart is the barcode of the eight target markers. (B) Histogram of the copy number of eight target markers detected in U87 cells by ProxISCA (cell number > 100). (C) Transmission electron microscopy images of amplicons. (D) Optimization of the design of proximity probes in U87 cells (cell number > 60). Statistical significance was determined by a two‐tailed *t*‐test: ***p < 0.001. (E) Relative expression levels of eight RNA markers in U87 cells. Data in this figure are mean ± s.d. (*n* = 3). (F) Visualization of eight target markers in U251 cells and MO3.13 cells by ProxISCA. (G) Single‐cell quantification of the average copy number of amplicons measured in U87, U251, and MO3.13 cells (cell number > 100). The color‐coding marks indicate the expression of markers matching molecular typing, and the red X indicates the almost unexpressed markers in molecular typing.

To verify the feasibility of ProxISCA for detecting multiple RNA mutations, we first performed the method on sGBM cell U87 for imaging eight RNA targets, including IDH 1wild type (WT), IDH1 mutation (MUT), ATRX WT, ATRX MUT, TERT WT, TERT MUT, 1p and 19q. The detailed coding assignments of the eight markers are described in Figure 1A. We decoded the amplicons through the fluorescence signals of the four channels and marked them with pseudocolors according to the coding assignment. The decoded pseudocolors were in good line with those in the initial merged image, suggesting that ProxISCA could precisely identify and encode multiple targets. We observed many separated superbright spots generated from targets, which were clearly distinct from the cellular background. Electrophoresis analysis demonstrated that the amplicons had a large size and could not transfer to agarose gels (Figure S1). Transmission electron microscopy (TEM) and dynamic light scattering (DLS) further presented that the amplified products were monodisperse particles with an average size of 295.00 nm (Figure 1C and Figure S2). This size can break the optical diffraction limit and can be resolvable by fluorescence microscopy. One‐target‐one‐amplicon amplification endowed this method with single‐molecule detection resolution. Next, we optimized the design of the proximity primers. The proximity primers simultaneously targeted RNA and locked loop probes, and we explored the sequence connecting the two targeted positions. The proximity probe for the longest hybridization length of the padlock probe and RNA presented a higher efficiency (Figure 1D).

Expression of IDH1 MUT (yellow dots), TERT MUT (light green dots), 1p‐D1S2666 (blue dots), 19q‐D1S412 (pink dots), and ATRX WT (red dots) can be observed in U87 cells (Figure 1A). We then counted the copy numbers of eight biomarkers in over 100 cells and observed evident variability in the copy numbers, suggesting that significant cellular expression heterogeneity was displayed even within the same batch of cells (Figure 1B and Figure S3). The average copy numbers of amplicons for IDH1MUT, TERT MUT, 1p‐D1S2666, 19q‐D1S412, and ATRX WT were 16.39, 10.63, 28.08, 11.41, and 16.17 per cell, respectively (Figure 1B,G). The results indicated that significant expression of 1p/19q with IDH1 mutation and TERT mutation existed in U87 cells, which is consistent with the molecular typing of human brain sGBM cells. ^[^ ^23^ ^]^ In addition, the expression profile of markers by ProxISCA was generally consistent with the RT‒qPCR results (Figure 1E).

Next, to validate that the fluorescent spot signals come solely from target RNA markers, we performed control experiments. From Figure S4, only a rare fluorescence signal could be seen when random probes were added or when the targeted sites in the RNA sequence were blocked with an unlabeled complementary probe before carrying out ProxISCA. Almost no fluorescent spots were observed when no padlock probe was used. Furthermore, we applied ProxISCA in several other cells for comparison, such as the sGBM cell line U251, oligodendrocytic cell line MO3.13 and HeLa cells. We found obvious expression of 1p/19q with IDH1 mutation and ATRX mutation but no TERT mutation in U251 cells (Figure 1F and Figure S5), which was in line with the molecular typing of sGBM cells. The loss of heterozygosity of 1p/19q was very obvious in MO3.13 cells (Figure S6). However, only a rare fluorescence signal was observed in HeLa cells (Figures S7 and S8), confirming that the biomarker expression in HeLa cells was not consistent with the glioma classification basis.^[^ ^23^, ^36^ ^]^ Our imaging results indicate that the florescent spots were not due to non‐specific effects. In addition, we observed different mutational statuses of TERT and ATRX in U251 and U87 cells (both sGBM cells) (Figure 1G). According to previous reports,^[^ ^23^ ^]^ IDH‐mutated sGBM with TERT MUT showed a better prognosis than that with ATRX MUT, indicating that U251 has a better prognosis than U87. All results suggest that ProxISCA can type cells by multiplex imaging RNA mutations.

### 3.3 Evaluation of specificity and accuracy of ProxISCA

**FIGURE 2:**
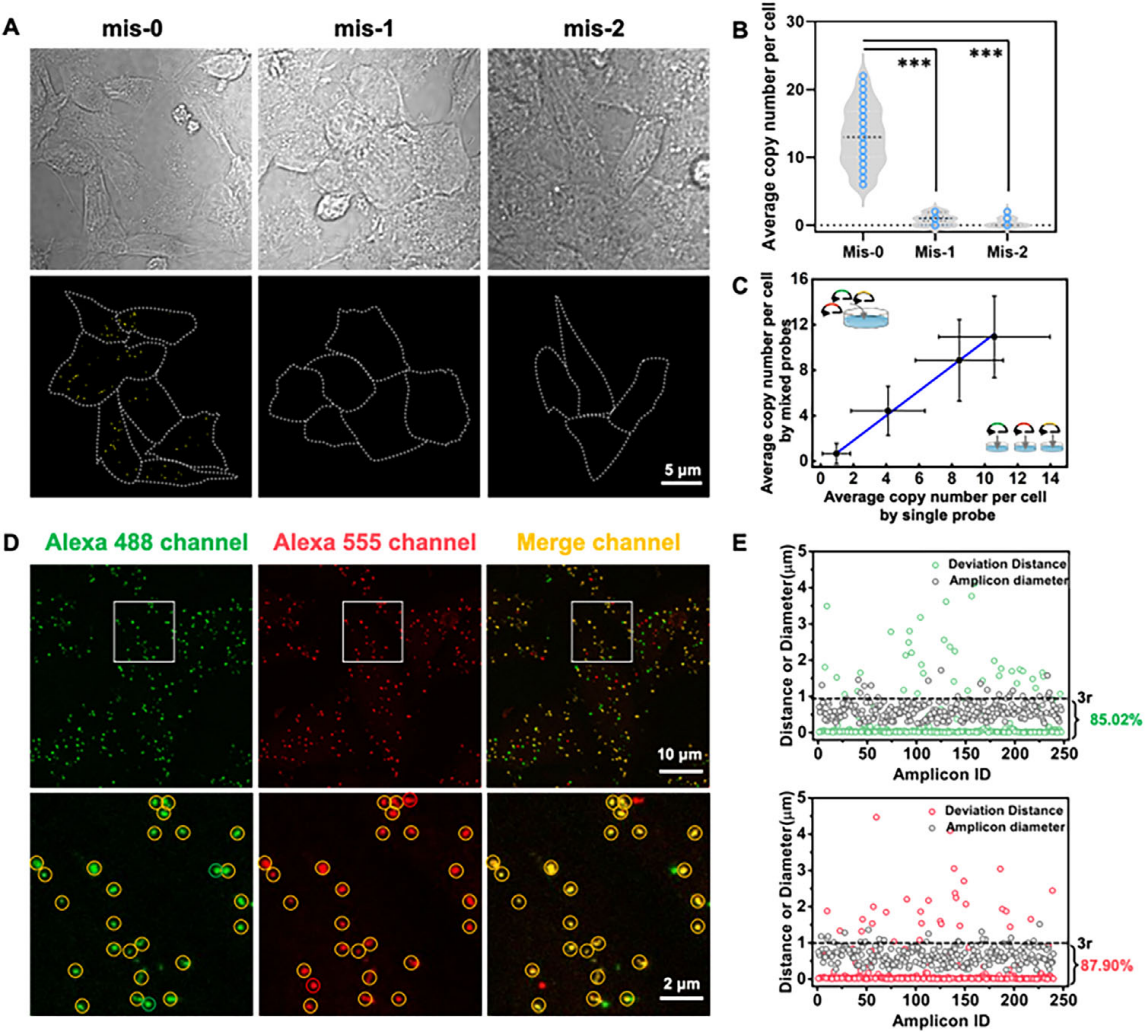
Specificity and accuracy analysis of ProxISCA for RNA mutation detection. (A) Confocal images of IDH1 MUT visualized by fully matched padlock probe (Mis‐0) and padlock probes altered by one (Mis‐1) or two bases (Mis‐2). Cells are outlined by gray solid lines, and yellow squares represent amplicons from IDH1 MUT. Scale bar: 5 μm. (B) Single‐cell quantification of the average copy number of amplicons measured in (A) (cell number > 100). Statistical significance is determined by a two‐tailed *t*‐test: *** represents the statistically significant differences between the two sets, ***p < 0.001. (C) Comparison of results by separated padlock probes with that of mixed padlock probes. (D) Colocalization signals at different sites on the same RNA by ProxISCA. The enlarged images are shown in the second row. The yellow circle indicates colocalized amplicons, and the green and red circles indicate amplicons that only appeared in the Alexa 488 channel or Alexa 555 channel, respectively. (E) The diameter of the spots in Alexa 488 (green) and Alexa 555 channels (red) and the deviation distance of fluorescent spots between the two channels in (D).

To test the specificity of the ProxISCA method, we altered the padlock probe targeting IDH1 by one or two bases. The measured single‐cell copy number dropped sharply after introducing one or two mismatched bases (Figure 2A). Almost no signal could be observed when using a padlock probe with one mismatched base (Mis‐1) or two mismatched bases (Mis‐2), while 13.39 copies per cell were detected by the fully matched padlock probe (Mis‐0) (Figure 2B). This method could specifically identify RNA mutations and discriminate RNAs with single‐base resolution.

To evaluate the accuracy of ProxISCA for multiplex imaging RNA mutations, we compared the expression levels of different RNA mutations measured by separated single padlock probes and mixed padlock probes. We gained a slope of 1.09 with R^2^ values of 0.999 for separated padlock probes in comparison with that of mixed padlock probes, demonstrating that there is little crosstalk between different targets when detected simultaneously (Figure 2C). To evaluate the spatial positioning accuracy, we performed a colocalization experiment in which the RNA was targeted at two sites. Two padlock probes with different fluorescent tag sequences were designed to target two regions of the target RNA. The fluorescent spots in Alexa 488 and 555 channels represented signals amplified at different sites on the same RNA. As illustrated in Figure 2D, clear colocalization signals (yellow spots) were presented in the merged channel. We then extracted the data of the positions and diameters of the bright spots and calculated the minimum distance between the bright spots in the two channels. Bright spots with a minimum distance of less than three times the radius were considered to be colocalized. The ratio of colocalization of amplicons in Alexa488 and Cy5 channels both were more than 85%, and the distance deviation of spots between the two channels was much less than the size of spots (Figure 2E). Thus, this method could precisely reveal the subcellular location of target mutations.

### 3.4 Demonstration of discriminating capacity of glioma cells by ProxISCA

**FIGURE 3:**
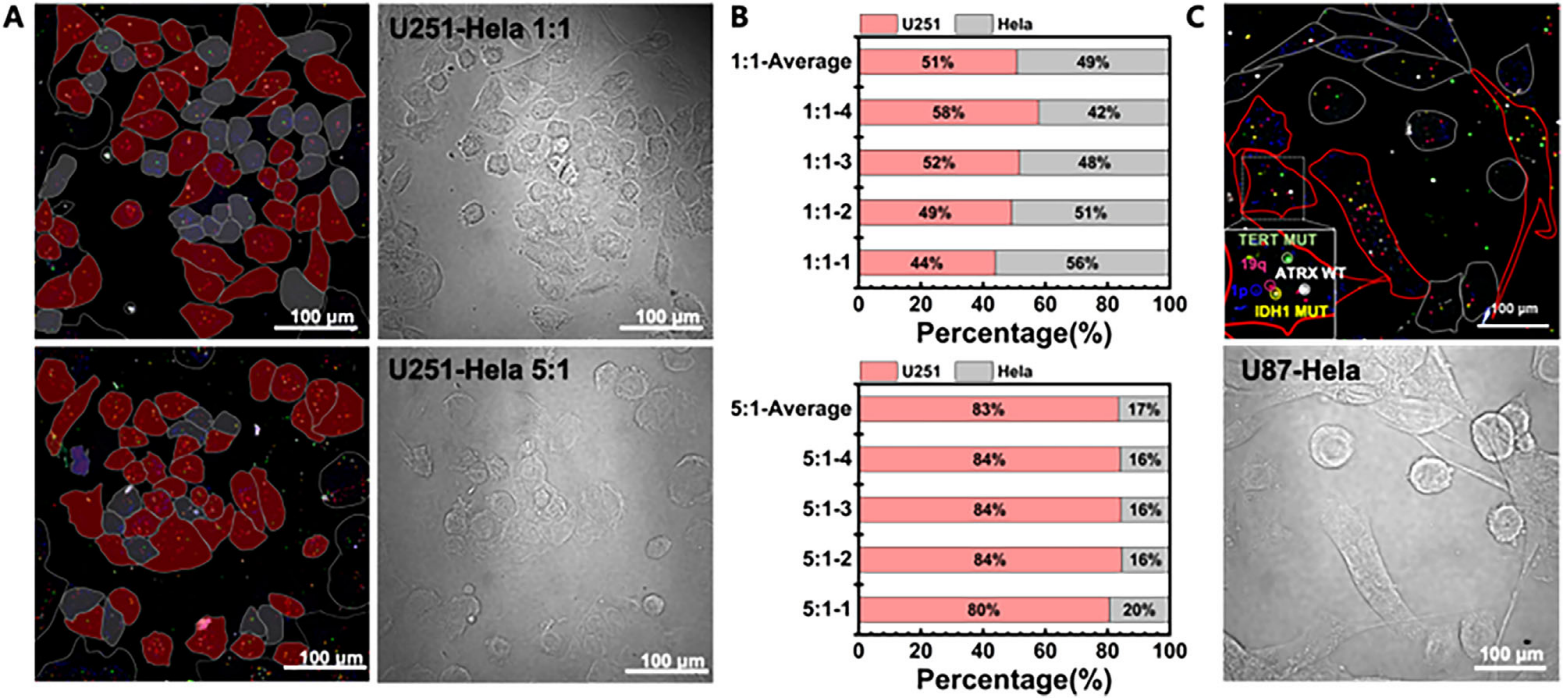
Discrimination of glioma cells from mixed cells by ProxISCA. (A) Fluorescence images of eight markers in mixed cells of U251 and HeLa by ProxISCA. The gray box line represents the cell area, the red area represents the U251 cell, and the gray area represents the HeLa cell. Scale bar: 100 μm. (B) Stacked histogram of the percentage of U251 and HeLa cells in the same confocal field. Four confocal fields were selected for different ratios (cell number > 100). (C) Confocal images of eight markers in mixed cells of U87 and HeLa cultured in random proportions. Inset: fluorescent image of the white box subregion with colored circles corresponding to the encoded RNA mutation molecules. The red and gray lines represent the cell outlines of the U87 cell and HeLa cell, respectively. Scale bar: 100 μm.

To assess the identification accuracy of ProxISCA for glioma cells, we first applied it to mixed cell types. We carried out a mixed culture of sGBM cells (U251) and control cells (HeLa) with cell number ratios of 1:1 and 5:1 and labelled the eight RNA mutation markers of mixed cells by ProxISCA. Figure 3A shows the expression and spatial distribution of target markers in U251 and HeLa cells under the same field of view. The red filled cells in Figure 3A are U251 cells, and the gray filled cells are HeLa cells based on marker gene typing. The number ratio of U251 cells and HeLa cells typed by ProxISCA was counted in Figure 3B. The results showed that the 1:1 ratio of cells was finally identified as 1.033:1, and the 5:1 ratio of cells was identified as 5.032:1. The ratio of the two cells identified by ProxISCA was similar to the preset cell mixing ratio.

We further verified the visual typing accuracy of this method by cell morphology. We selected U87 and HeLa cells with large differences in cell morphology for mixed coculture at random concentrations. From Figure 3C, the confocal brightfield image clearly shows the difference between the two cell morphologies. U87 cells are long in shape and have synaptic‐like endings, while HeLa cells are spindle‐shaped or irregular polygonal‐shaped. There were also significant differences in RNA marker expression in the fluorescence field images. The complete expression of 1p and 19q, as well as the expression of IDH MUT with TERT MUT or ATRX MUT, was observed in U87 cells, while the expression of markers in HeLa cells was not consistent with molecular typing basis of sGBM. The identification of U87 cells in mixed culture cells by ProxISCA is basically consistent with the results of cell morphology identification. All these results indicated that ProxISCA can precisely discriminate glioma cells.

### 3.5 Visual typing of gliomas with different pathological grades at the tissue level

**FIGURE 4:**
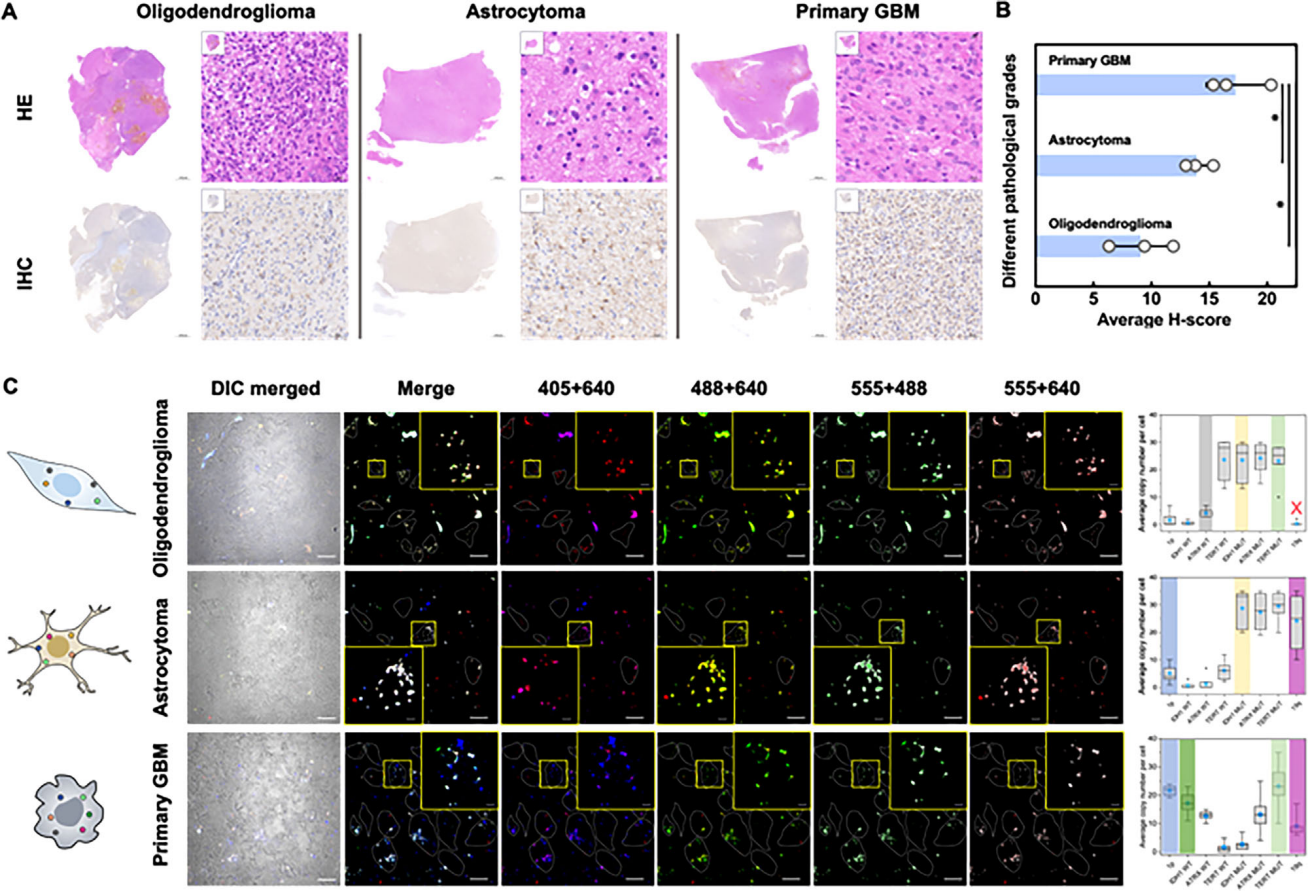
Visual typing of glioma tissues with different pathological grades. (A) HE staining and GFAP immunohistochemical staining of oligodendroglioma, astrocytoma, and primary GBM. (B) Average H‐score of three types of glioma tissues. * indicates statistically significant differences between the two groups, *p < 0.05. Data in (B) are mean ± s.d. (*n* = 3). (C) The right is a conceptual diagram of the distribution of eight targets in oligodendroglioma, astrocytoma, and primary GBM tissue sections. In the middle are confocal images of eight RNA markers in the tissue sections visualized by ProxISCA. Merged channels of 405 and 640 represent the signal of 19q (magenta). Merged channels of 488 and 640 represent IDH1 MUT (yellow). Merged channels of 555 and 488 represent TERT MUT (light green). Merged channels of 555 and 640 channels represent ATRX MUT (light red). On the left is single‐cell quantification of the average copy number of amplicons measured in the middle (cell number > 100). The color‐coding marks indicate the expression of markers matching molecular typing, and the red X indicates the almost unexpressed markers in molecular typing.

We next applied ProxISCA to image RNA mutation markers in glioma tissue sections from patient samples with different pathological grades. The tissue sections were first characterized by traditional hematoxylin and eosin (H&E) staining (Figure 4A). In the oligodendroglioma tissue section, the perinucleus was white, and the nuclei were evenly distributed. Astrocytoma tissue is composed of astrocytes of different sizes, and the cell density is lower than that of oligodendroglioma tissue. In contrast, primary GBM tissue is significantly multinucleated.^[^ ^37^, ^38^ ^]^ Immunohistochemistry (IHC) analysis was further performed to verify the pathological grade and malignant development of glioma.^[^ ^39^ ^]^ Figure 4B confirmed that the expression of GFAP protein was gradually increased in the tissue sections of oligodendroglioma, astrocytoma, and primary glioblastoma. From the fluorescence image results (Figure 4C), three gliomas exhibited distinct expression profiles. Only primary GBM showed obvious expression of IDH1 WT, and only oligodendroglioma presented the deletion of 19q. In addition, three gliomas showed expression of ATRX MUT or TERT MUT. The genetic expression profiles of different glioma tissue sections were in accordance with the molecular typing basis. Briefly, the results of ProxISCA are consistent with the pathologic H&E and IHC observations of tumor tissues. Moreover, the approach can provide accurate quantification and spatial location of RNA markers, as well as single‐cell heterogeneity information.

### 3.6 Heterogeneity analysis of glioma RNA markers

**FIGURE 5:**
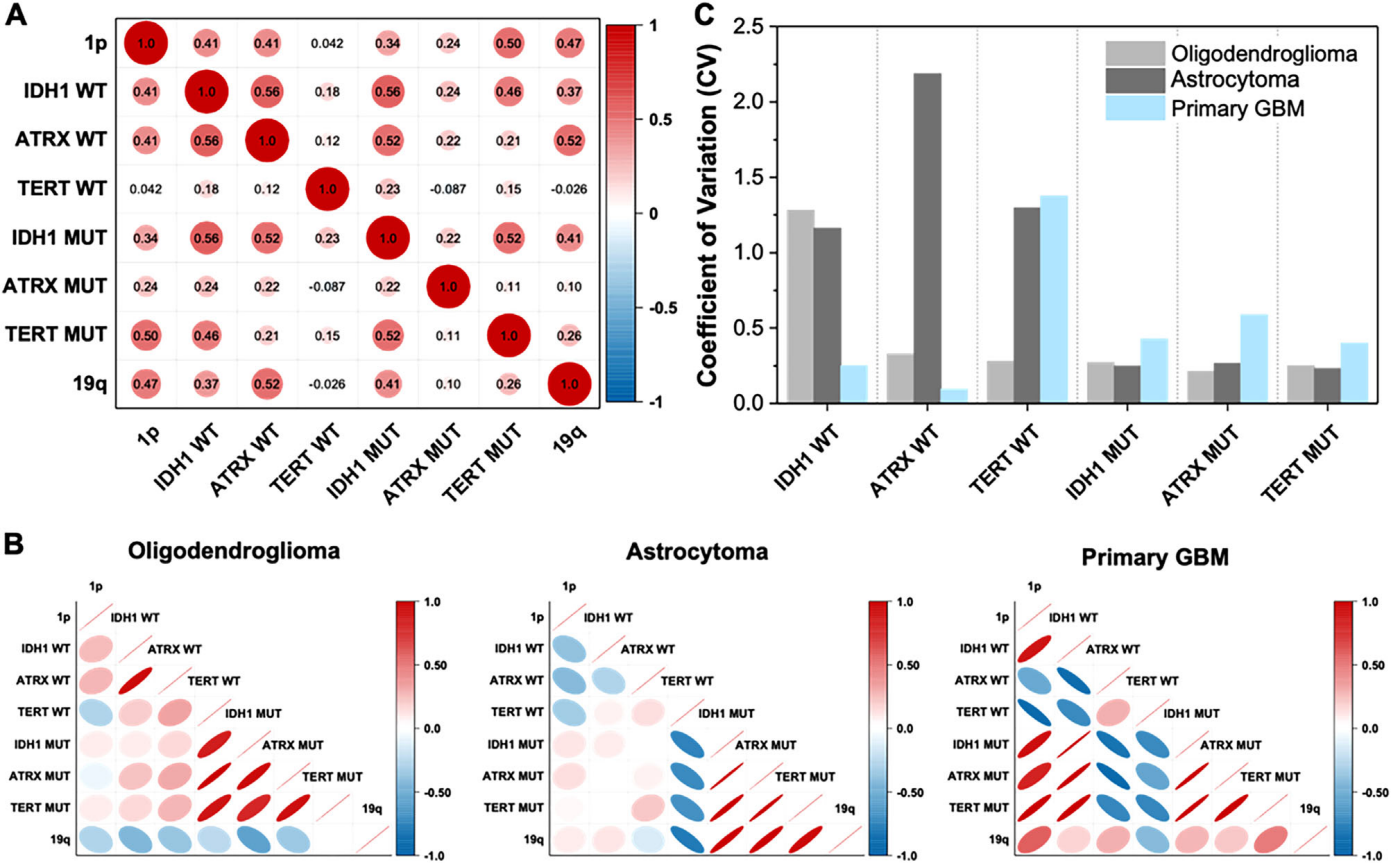
Heterogeneity analysis of glioma RNA markers. Pairwise correlation coefficient matrix of cell‐to‐cell expression variation for eight RNA markers in (A) U87 cells and glioma tissues with (B) different pathological grades. The correlation matrix was done using Origin 9.1 software (OriginLab, USA). (C) Coefficient of variation of gliomas with different pathological grades.

ProxISCA's capacity for parallel profiling of multiple targets at the single‐cell level enables investigating gene expression correlation and heterogeneity. We then explored the expression covariation among RNA markers, which might be caused by the differential activity of malignant development. The greater the absolute value of the correlation coefficient, the stronger the correlation. As illustrated in Figure 5A, Figures S9, S10 and Table S3, almost all the pairwise correlation coefficients between RNA markers in MO3.13 cells were small (<0.2), indicating no significant correlation. In U87 sGBM cells, 12 pairs of markers showed moderate correlation (0.4–0.6). Among them, the correlations between TERT MUT and IDH1 WT, TERT MUT and IDH1 MUT, and 1p and TERT MUT have been reported in previous works.^[^ ^40^, ^41^, ^42^ ^]^ This indicates that U87 cells express more genes with high correlation than MO3.13 cells. We also found more correlated markers expressed (correlation coefficients > 0.6) in primary GBM tissue (18 pairs) than in astrocytoma (10 pairs) and oligodendroglioma tissues (7 pairs) (Figure 5B). The results indicated that gliomas with high malignant grade express more genes with high correlation at the cellular and tissue levels. Correlation analysis can be used to infer gene functions and regulatory pathways.

We also calculated the coefficients of variation (CV, SD/mean) of different tissues (Figure 5C). It was found that the RNA markers of oligodendroglioma presented the lowest CV values except for IDH1 WT. The CV values of most RNA markers (such as TERT WT, IDH1 MUT, ATRX MUT, and TERT MUT) of primary GBM were greater than those of the corresponding genes of oligodendroglioma and astrocytoma. The results suggest that glioma with a higher malignant grade has greater cell heterogeneity, and this heterogeneity may reflect functional differences at the pathological level.

## 4 CONCLUSION

We have developed a multiplexed imaging method of RNA mutations, termed ProxISCA, to enable visual typing of brain gliomas with different pathological grades at the single‐cell and tissue levels. To our knowledge, this study is the first multiplexed and spatial analysis of RNA mutation patterns from glioma patients. This imaging method has several merits: (1) The ligation‐based padlock probe can discriminate one‐nucleotide variations, and the proximity primer can prevent the amplified product from falling off the target and improve the accuracy of spatial location. (2) The DNA module‐based spectral encoding strategy can enhance detection throughput through just one round of labelling, break through the limitation of fluorescence spectral overlap, and has programmability and extensibility. In addition, this method significantly reduces the cost of probe synthesis due to the utilization of only a few specific spectrally labelled probes. Multimutation biomarker detection potentially improves typing accuracy. (3) The one‐target‐one‐amplicon amplification endows the method with single‐molecule resolution. The high amplification signal could reduce background interference from tissue complexity, and the mild and isothermal conditions contribute to maintaining cell or tissue structure and morphology.

Moreover, compared to sequencing‐based techniques, ProxISCA adds an additional dimension besides mutation information to glioma research by acquiring cellular heterogeneity information and revealing the subcellular location of RNA mutations and even the spatial localization of glioma cells in brain tissue. We have found that gliomas with high malignant grade express more genes with high correlation at cellular and tissue levels, and cellular heterogeneity was positively correlated with glioma malignancy, which has not been discovered by previous cell‐population‐based methods. This method also has some limitations. For instance, the imaging throughput of ProxISCA is still limited, and it is difficult to detect hundreds of RNA mutations like the single‐cell sequencing approaches. Besides, this method has relatively low in situ detection efficiency, which is particularly evident in tissue imaging. In the future, the throughput can be improved by constructing a two‐dimensional encoding mechanism. For example, the fluorescence intensity dimension can be introduced based on DNA module coding. In addition, the imaging efficiency would be improved by optimizing the secondary structure of the padlock probe, increasing tissue penetration of probes, or imaging depth, such as optical tissue clearing. In conclusion, ProxISCA can not only achieve in situ typing of glioma cells in brain tissue but also reveal the precise connection between heterogeneous cell behavior and the occurrence/development of glioma, which benefits personalized therapy, precise treatment, and the prognosis of glioma patients.

## CONFLICT OF INTEREST STATEMENT

The authors declare no conflicts of interest.

## Supporting information

Supporting Information

## Data Availability

Supporting Information is available from the Wiley Online Library or from the author.
