# Supplementary material for: Visual genetic typing of glioma using proximity‐anchored in situ spectral coding amplification
Source: Exploration (Beijing). 2023 Jul 6;3(5):20220175. doi: 10.1002/EXP.20220175 (PMC10582607; doi:10.1002/EXP.20220175)
Supplement: Supplementary file 1 — Supporting Information [file EXP2-3-20220175-s001.docx]

**Supporting information**

# Visual genetic typing of glioma using proximity anchored in situ spectral coding amplification

Xiaolei Chen^#a^, Ruijie Deng^#b^, Dongdong Su*^a^, Xiaochen Ma^a^, Xu Han^e^, Shizheng Wang^a^, Yuqing Xia^a^, Zifu Yang^a^, Ningqiang Gong^d^*,* Yanwei Jia^c^, and Xiaojun Ren*^a^, Xueyun Gao*^a^

^a^ Department of Chemistry and Biology, Faculty of Environment and Life Science, Beijing University of Technology Beijing 100124 (China)

^b^ College of Biomass Science and Engineering, Healthy Food Evaluation Research Center, Sichuan University, Chengdu 610065, China

^c^ State-Key Laboratory of Analog and Mixed-Signal VLSI, Institute of Microelectronics, University of Macau, Macau, China.

^d^ Department of Bioengineering, University of Pennsylvania, Philadelphia, PA, USA.

^e^ Institute of High Energy Physics, Chinese Academy of Sciences, Beijing, China

^#^Xiaolei Chen and Ruijie Deng contributed equally to this work.

*Correspondence:* [*xjren@bjut.edu.cn*](mailto:xjren@bjut.edu.cn)*;* gaoxy@ihep.ac.cn; [*chmsudd@bjut.edu.cn*](mailto:chmsudd@bjut.edu.cn)*;*

# Oligonucleotide sequences

**Table S1.** DNA Sequences for SnailTIEA

| Name | Sequence（5’-3’） | Description |
| --- | --- | --- |
| IDH1 WT | CGACCTATGATGATATTTTAGTGCGAACTACTACTCTCTCTTTTTAGTGCGAACTACTACTCTCTCTGGGTATTTCCCCATAAGCATGA | DNA sequences for in vitro RCA - Padlock probe  [a] The underlined sequences are complementary to the RNA sequences.  [b] The sequences marked in blue are the complementary sequences of Primer 405 and MB 405.  [c] The sequences marked in green are the complementary sequences of Primer 488 and MB 488.  [d] The sequences marked in orange are the complementary sequences of Primer 555 and MB 555.  [e] The sequences marked in red are the complementary sequences of Primer 640 and MB 640.  [f] Bases labeled in bright undertones are specific for identifying mutation sites. |
| IDH1 MUT | TGACCTATGATGATATTTTAGTGCGAACTACTACTCTCTCTTTTTTGCGTCTATTTTCTGGAGCCATGGGTATTTCCCCATAAGCATGA |  |
| ATRX WT | ACTGTGACAGGCACTTTTTCTCAATTCTGCTACTGTACTACTTTTCTCAATTCTGCTACTGTACTACTTTTTCATCATCATCATCC |  |
| ATRX MUT | TCTGTGACAGGCACTTTTTCTCAATTCTGCTACTGTACTACTTTTTGCGTCTATTTTCTGGAGCCATTTTTTCATCATCATCATCC |  |
| TERT WT | GCAGTTTTGACTCAGTTTTTGCGTCTATTTTCTGGAGCCATTTTTTGCGTCTATTTTCTGGAGCCATTTTTTATCCCATGGAGGTG |  |
| TERT MUT | ACAGTTTTGACTCAGTTTTAGTGCGAACTACTACTCTCTCTTTTTCTCAATTCTGCTACTGTACTACTTTTTATCCCATGGAGGTG |  |
| 1p-D1S2666 | ATATTTTCTTGTAGTTTTTAACTATACAACATACTACCTCATTTTAACTATACAACATACTACCTCATTTTTGAAGCATTTCCCCT |  |
| 19q-D1S412 | ATCCGCACCATTGCATTTTAACTATACAACATACTACCTCATTTTTGCGTCTATTTTCTGGAGCCATTTTTTTGCAGTGAGCCGAG |  |
| IDH1 MUT mis-0 | TGACCTATGATGATATTTTAGTGCGAACTACTACTCTCTCTTTTTTGCGTCTATTTTCTGGAGCCATGGGTATTTCCCCATAAGCATGA | Sequences for specificity analysis experiments - IDH1 MUT base mismatch sequence.  [a] The bases marked in red are the mismatch positions. |
| IDH1 MUT mis-1 | AGACCTATGATGATATTTTAGTGCGAACTACTACTCTCTCTTTTTTGCGTCTATTTTCTGGAGCCATGGGTATTTCCCCATAAGCATGA |  |
| IDH1 MUT mis-2 | AGACCTATGATGATATTTTAGTGCGAACTACTACTCTCTCTTTTTTGCGTCTATTTTCTGGAGCCATGGGTATTTCCCCATAAGCATGT |  |
| IDH1 WT（NM_005896.4） | TATCATCATAGGTC**G**TCATGCTTATGGGGA | Targeted oligonucletotide sequences of eight RNA markers.  The bases marked in red are mutation sites. The target sequences were selected from the mutation site before and after a total of 30 nts. |
| ATRX WT（AB102641.1） | AGTGCCTGTCACAG**T**GGATGATGATGATGA |  |
| TERT WT（AF097365.1） | CTGAGTCAAAACTG**C**CACCTCCATGGGATA |  |
| 1p-D1S2666 （Z52571.1） | ACTACAAGAAAATATAGGGGAAATGCTTCA |  |
| 19q-D1S412（Z23298.1） | TGCAATGGTGCGGATCTCGGCTCACTGCAA |  |
| Proximity primer-IDH1 | GGTTTTACCCATCCAAAAAAGAGAGAGTAGTAGTT | Proximity primer sequence used in ProxISCA |
| Proximity primer-ATRX | GATTTAGATAAGGCTAAAAGTAGTACAGTAGCAGA |  |
| Proximity primer -TERT MUT | CGTAATTCCCTTGAGAAAAAGAGAGAGTAGTAGTT |  |
| Proximity primer -TERT WT | CGTAATTCCCTTGAGAAAAATGGCTCCAGAAAATA |  |
| Proximity primer-1p | TGTGTGTGTGTGTGTAAAATGAGGTAGTATGTTGT |  |
| Proximity primer 19q | TCAAAAAGAAAAAAAAATGAGGTAGTATGTTGTAT |  |
| primer 405 | TGAGGTAGTATGTTGTATAGTT | Primer sequences used for in vitro amplification |
| primer 488 | AGAGAGAGTAGTAGTTCGCACT |  |
| primer 555 | GTAGTACAGTAGCAGAATTGAG |  |
| primer 640 | ATGGCTCCAGAAAATAGACGCA |  |
| MB 405 | AACTATACAACATACTACCTCA | DNA sequences for in vitro RCA – RCA detection probes |
| MB 488 | AGTGCGAACTACTACTCTCTCT |  |
| MB 555 | CTCAATTCTGCTACTGTACTAC |  |
| MB 640 | TGCGTCTATTTTCTGGAGCCAT |  |

**Table S2.** DNA sequences for RT-qPCR analysis

| qPCR primer sequences | Sequence（5’-3’） |
| --- | --- |
| GAPDH-F | CACCATGGGGAAGGTGAAGG |
| GAPDH-R | CCACCTGGTGCTCAGTGTAG |
| IDH1WT-F | GGTAAAACCTATCATCATAGGTCG |
| IDH1MUT-F | GGGTAAAACCTATCATCATAGGTCA |
| IDH1-R | TTAGACAGAGCCATTTGGAAGGA |
| ATRX WT-F | ATCTAAATCAGTGCCTGTCACAGT |
| ATRX MUT-F | ATCTAAATCAGTGCCTGTCACAGA |
| ATRX-R | GCTCCGCTGATTTTCTTCCAACTC |
| TERT WT-F | TTACGCTGAGTCAAAACTGC |
| TERT MUT-F | TTACGCTGAGTCAAAACTGT |
| TERT-R | GCACTTCAGCCCTGTCCTTGGATG |
| 1p-D1S2666-F | ATTGCAGCCTGGTCAAGAAGAGCG |
| 1p-D1S2666-R | CCTTACCCAGACCAATATCATG |
| 19q-D1S412-F | TCCACAGTCATTTGAGTCCTTCC |
| 19q-D1S412-R | CTAGAGAAGCAGAGCCAATAGC |

**Table S3.** The distribution of gene pairwise correlation coefficients for different cell / tissue types

| **Types** | **Number of pairs of markers** | | | |
| --- | --- | --- | --- | --- |
|  | <0.2 | 0.2-0.4 | 0.4-0.6 | >0.6 |
| U87 | 8 | 8 | 12 | 0 |
| MO3.13 | 27 | 1 | 0 | 0 |
| Oligodendroglioma | 7 | 12 | 2 | 7 |
| Astrocytoma | 13 | 4 | 5 | 10 |
| Primary GBM | 1 | 4 | 5 | 18 |

^[a]^ > 0.6: strong correlation; 0.4-0.6: moderate correlation; 0.2-0.4: weak correlation; < 0.2: very weak correlation or no correlation


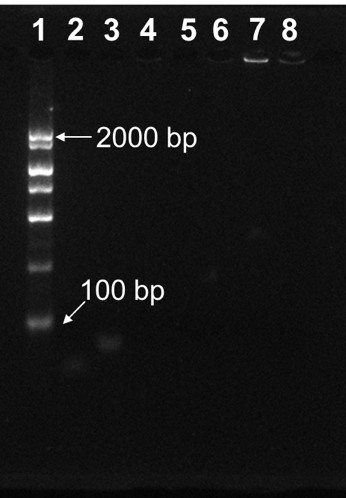


**Figure S1.** Characterization of molecular weight of amplification products - agarose gel electrophoresis. Lane 1: Marker. Lane 2: Only Target. Lane 3: Only padlock probe. Lane 4: No phi29 DNA polymerase. Lane 5: No T4 ligase. Lane 6: Padlock probe-mis-2. Lane 7: Padlock probe-mis-0. Lane 8: Padlock probe-mis-1. Only lane 7 can trigger the RCA reaction to produce products of large molecular weight that remain in the pore.


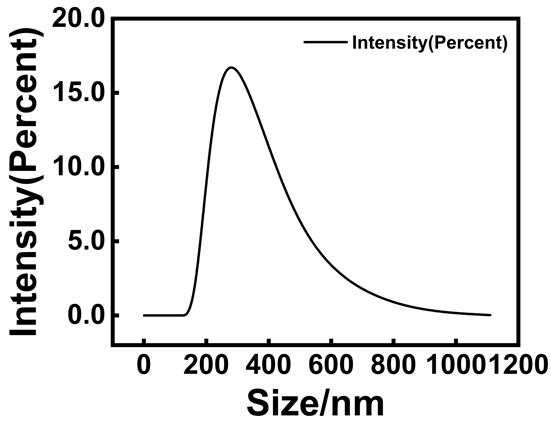


**Figure S2.** Measurement of diameter size and distribution of amplicons by dynamic Light scattering.

**
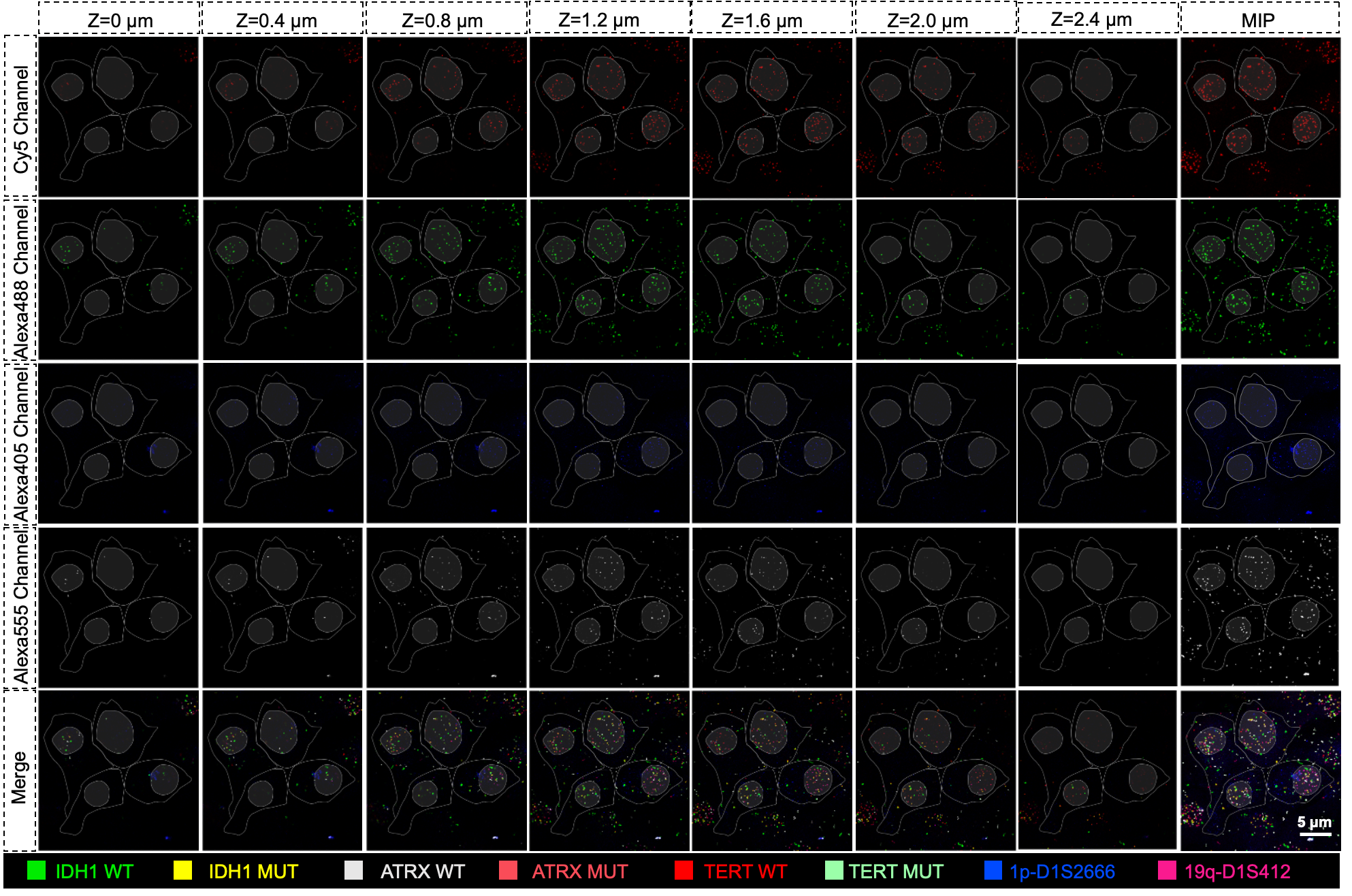
**

**Figure S3.** The z axis slices of confocal fluorescence images for 8 biomarkers imaged by ProxISCA in Alexa405, Alexa488, Alexa555, Cy5, merged and DIC merged channels. The outlines of cell and nucleus are marked by a gray line. Scale bars: 5 μm.

*
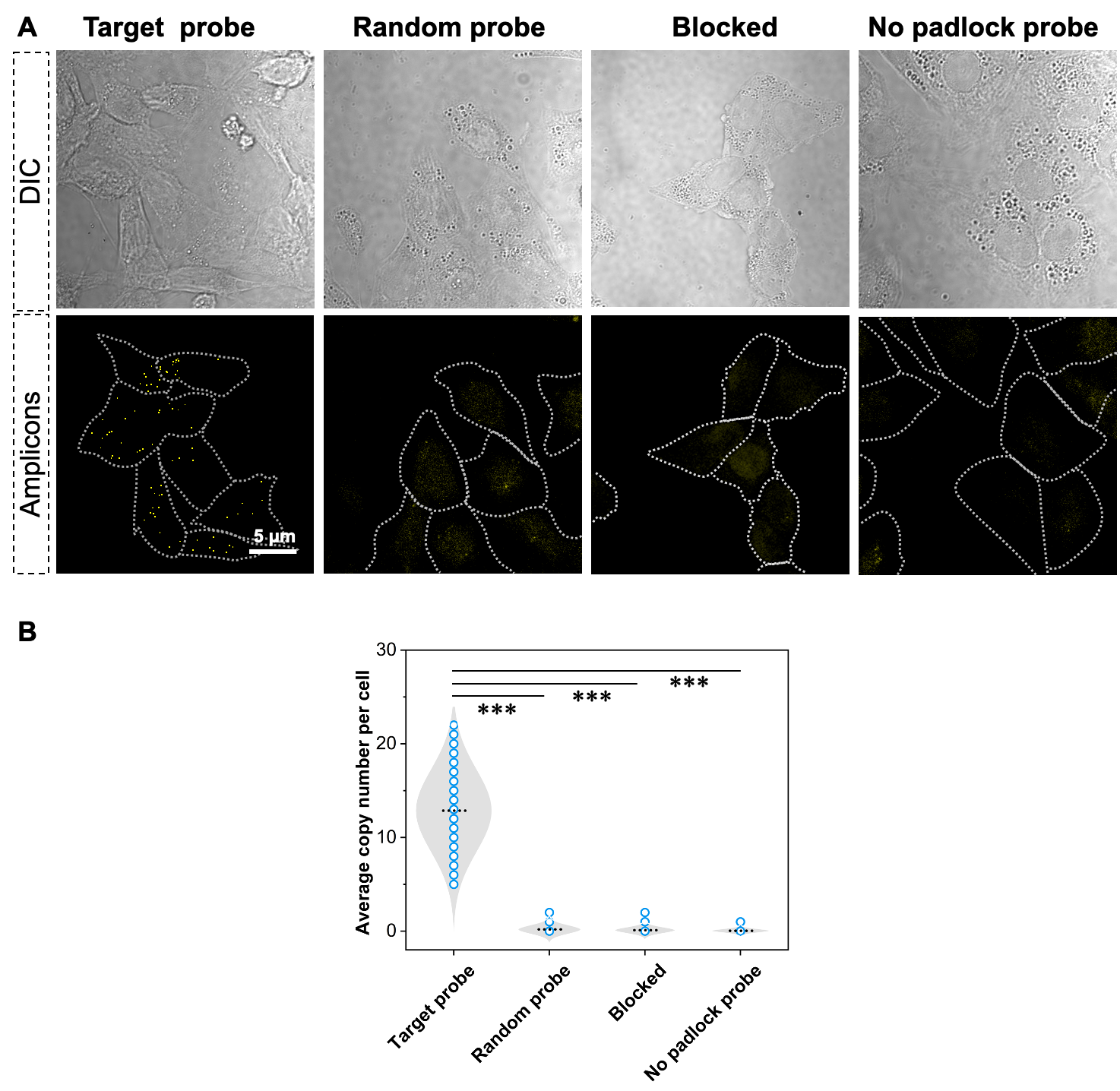
*

**Figure S4.** The fluorescence images and DIC images of IDH1 MUT in U87 cells imaged by SpliceRCA under different conditions: using target padlock probe, using random padlock probe, after blocking the target sites and without padlock probe. The yellow spots represent amplicons hybridized with Alexa555-labeled detection probes, the blue spots represent RCA amplicons hybridized with Cy5-labeled detection probes, and the outline of U87 cells is marked in a gray dot line. Scale bars: 5 μm; B) Quantification of the average number of amplicons per cell detected under above conditions.


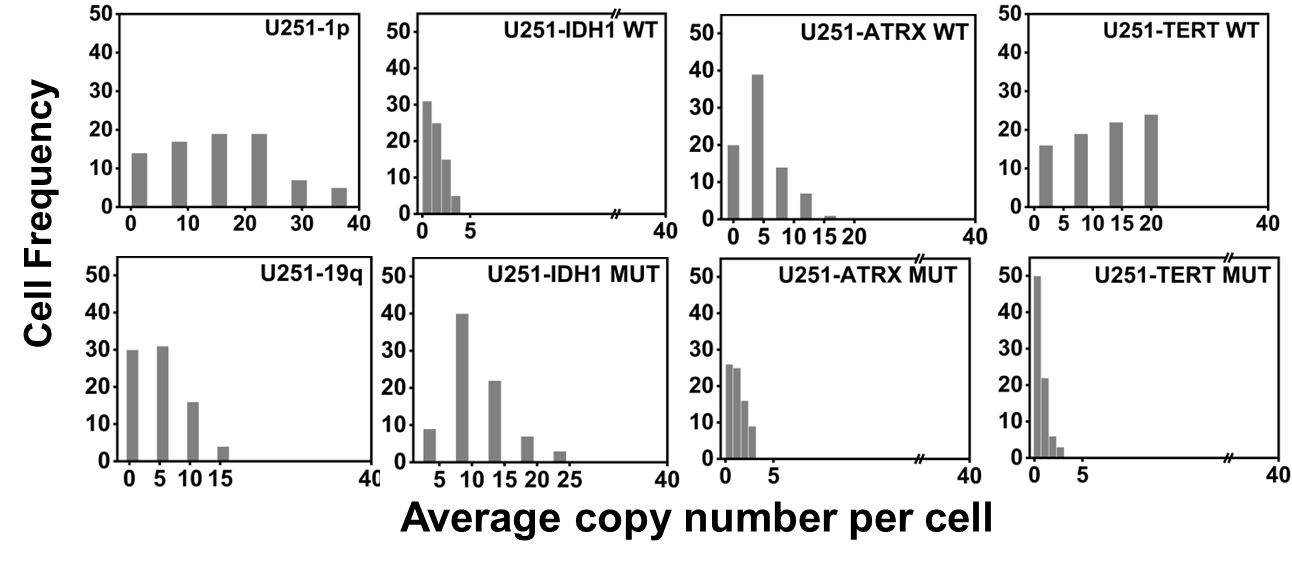


**Figure S5.** Single-cell frequency statistics of eight RNA markers in U251 cells (Cell number>100).


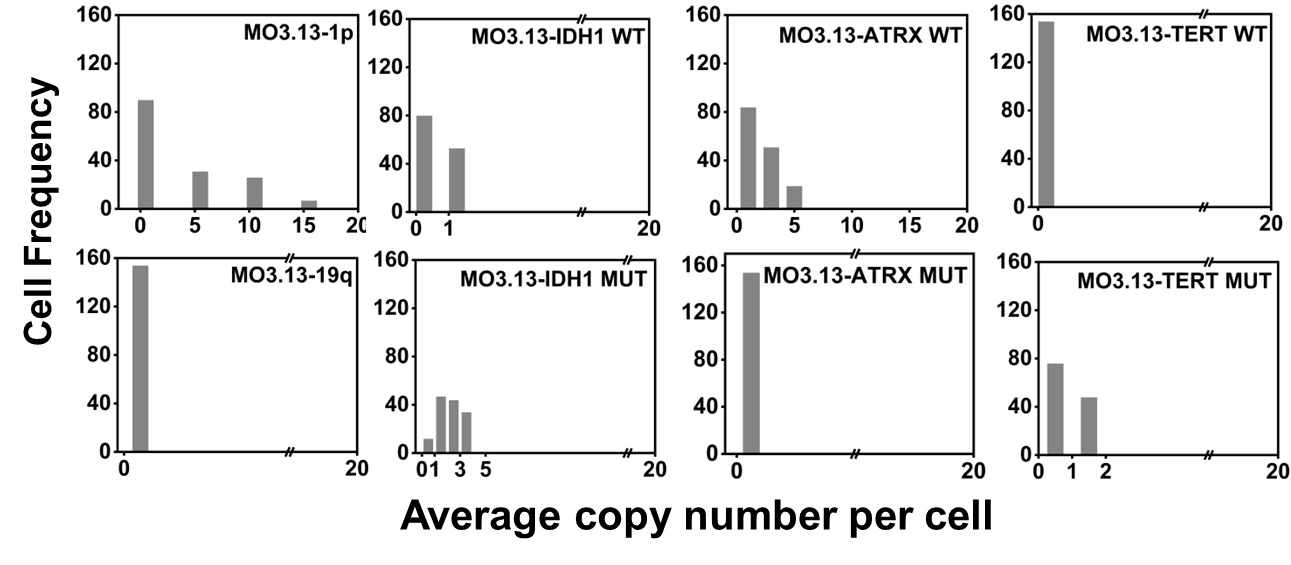


**Figure S6.** Single-cell frequency statistics of eight RNA markers in MO3.13 cells (Cell number>100).


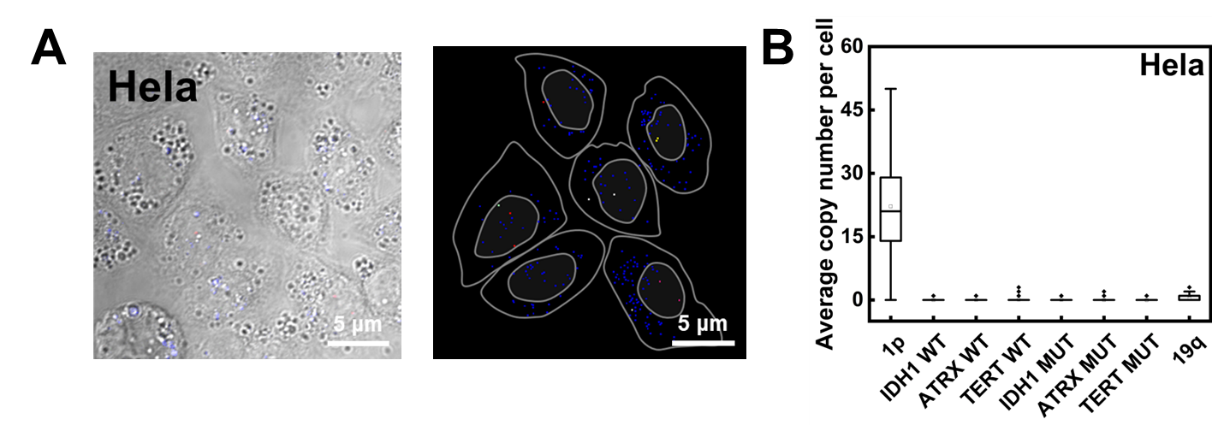


**Figure S7.** Fluorescence images (A) and quantitative analysis (B) of target markers in HeLa cells.


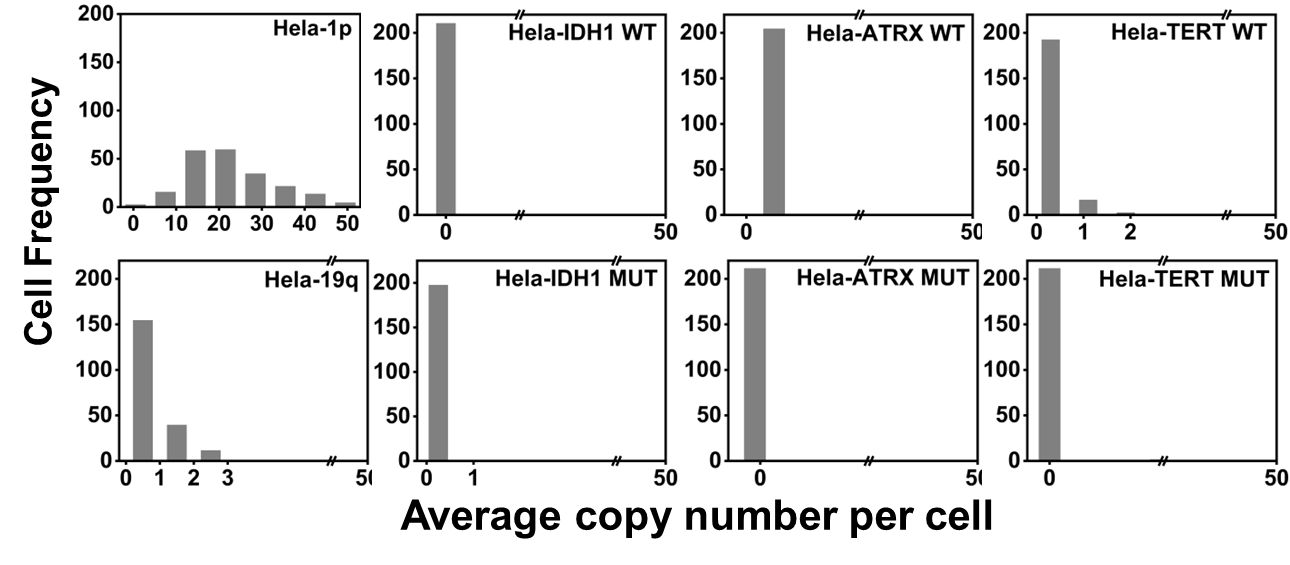


**Figure S8.** Single-cell frequency statistics of eight RNA markers in Hela cells (Cell number>100).


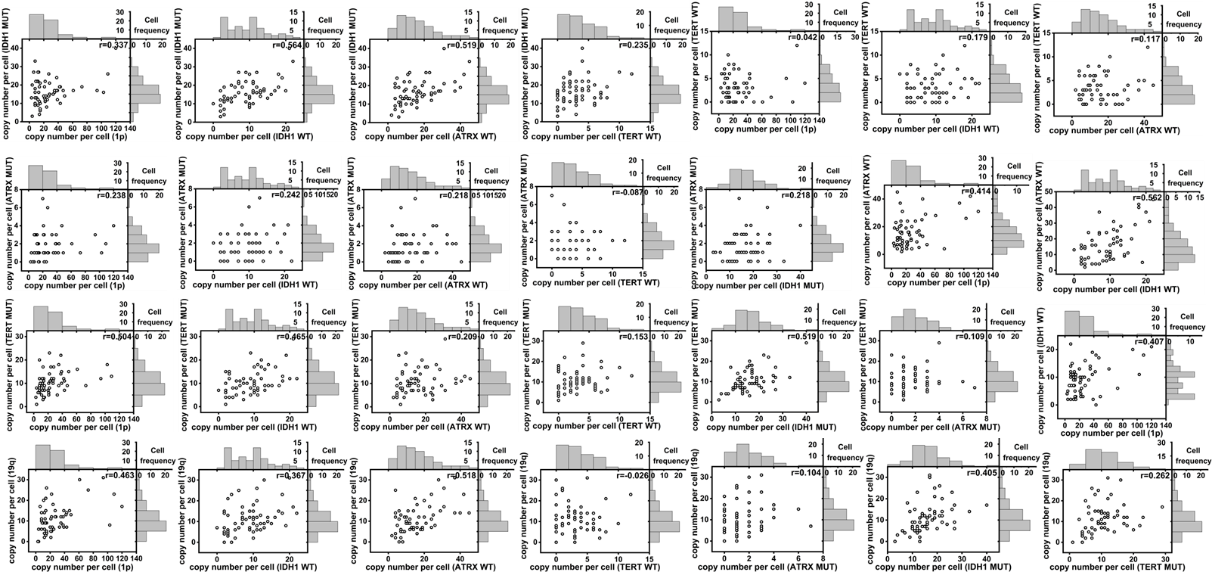


**Figure S9.** Correlation and marginal distributions of eight target markers in U87MG cells. r is the Pearson correlation coefficient.


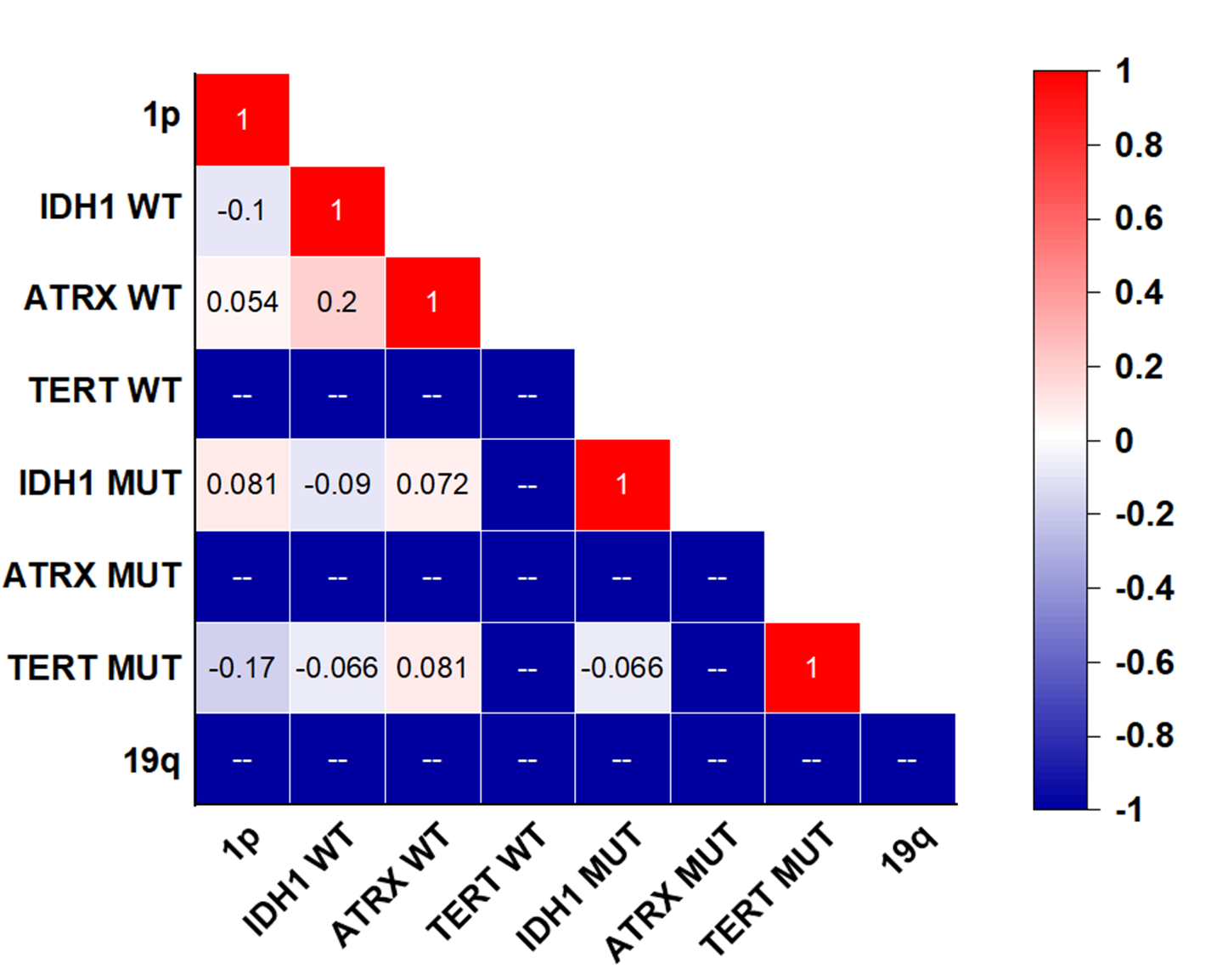


**Figure S10.** Pairwise correlation coefficient matrix of cell-to-cell expression variation for eight target RNA mutations in MO3.13 cells.
